# Coseismic fault-slip distribution of the 2019 Ridgecrest Mw6.4 and Mw7.1 earthquakes

**DOI:** 10.1038/s41598-021-93521-0

**Published:** 2021-07-09

**Authors:** Yang Gao, HuRong Duan, YongZhi Zhang, JiaYing Chen, HeTing Jian, Rui Wu, WenHao Yin

**Affiliations:** 1 College of Geological Engineering and Geomatics, Chang’an University, Xi’an, 710064 Shaanxi China; 2 College of Geomatics, Xi’an University of Science and Technology, Xi’an, 710054 Shaanxi China; 3 Xi’an Institute of Surveying and Mapping, Xi’an, 710054 Shaanxi China

**Keywords:** Solid Earth sciences, Geodynamics, Geology, Geophysics, Seismology, Tectonics

## Abstract

The 2019 Ridgecrest, California seismic sequence, including an Mw6.4 foreshock and Mw7.1 mainshock, represent the largest regional seismic events within the past 20 years. To obtain accurate coseismic fault-slip distribution, we used precise positioning data of small earthquakes from January 2019 to October 2020 to determine the dip parameters of the eight fault geometry, and used the Interferometric Synthetic Aperture Radar (InSAR) data processed by Xu et al. (Seismol Res Lett 91(4):1979–1985, 2020) at UCSD to constrain inversion of the fault-slip distribution of both earthquakes. The results showed that all faults were sinistral strike-slips with minor dip-slip components, exception for dextral strike-slip fault F2. Fault-slip mainly occurred at depths of 0–12 km, with a maximum slip of 3.0 m. The F1 fault contained two slip peaks located at 2 km of fault S4 and 6 km of fault S5 depth, the latter being located directly above the Mw7.1hypocenter. Two slip peaks with maximum slip of 1.5 m located 8 and 20 km from the SW endpoint of the F2 fault were also identified, and the latter corresponds to the Mw6.4 earthquake. We also analyzed the influence of different inversion parameters on the fault slip distribution, and found that the slip momentum smoothing condition was more suitable for the inversion of the earthquakes slip distribution than the stress-drop smoothing condition.

## Introduction

According to seismic information from the United States Geological Survey (USGS), an Mw6.4 earthquake occurred near Ridgecrest, California, on July 4, 2019 at 17:33 (UTC). The focal depth was 10.5 km and the movement on the fault was sinistral strike-slip. Approximately 34 h later, an Mw7.1 earthquake occurred 10 km northwest along the direction of the aftershock distribution. Its focal depth was 8.0 km and movement on the fault was dextral strike-slip^1^. The focuses of the earthquakes had a similar locations and the seismic interval was short.

These two earthquakes occurred near the Little Lake and Airport Lake fault zones, both of faults have a long history of activity^2^. Together,the spatial distribution of seismicity and focal plane solutions, as well as the surface ruptures, indicate that the strikes of the Mw6.4 and Mw7.1 faults were nearly orthogonal^2,3^. This type of complex geometrical structure fault is widespread. Previous records of conjugate faults include the 1992 Big Bear and Landers earthquakes in California^4^, 2010 Darfield Mw7.1 earthquake in New Zealand^5,6^, 2010–2011 seismic sequence in Rigan^7,8^, and 2018 Gulf of Alaska Mw7.9 earthquake^9,10^. The orthogonality of the two main faults and the effects of their seismic interaction have attracted extensive attention.

We found that the fault geometry, inversion data and constraint conditions affect the inversion results. Previous researcher has divided such faults into three sub-faults^11^, six sub-faults^12^, seven sub-faults^13^, or eight sub-faults^14^. The Ridgecrest Mw6.4 and Mw7.1 earthquake caused ground fractures, whose alignment on the ground surface can be discerned from WorldView satellite images^15^. In addition, a large number of small earthquakes occurred near the fracture, providing basic information for determining the geometric parameters^16,17^. Improvements in seismic positioning technologies have made it possible to acquire large amounts of accurate small-earthquake data that can aid in establishing fault dip using seismic clustering^18–20^. Li et al.^14^ used GPS data to obtain fault dip Angle parameters through geodetic inversion, and Feng et al.^13^ used InSAR data to obtain fault dip angle parameters through geodetic inversion. Both processes were carried out under the assumption of uniform slip on the fault surface. As for the researches on 2019 Ridgecrest Earthquakes, previous scholars have not determined the dip angle from the aspect of earthquake cluster. In this paper, the clustering property of small earthquakes was used to determine the dip angle of faults without assumption of uniform slip on the fault surface.

In previous studies, the main types of data used include InSAR, seismic waves, GPS, and data of strong surface vibrations^11–14^. Among these, GPS data points are relatively sparse in seismic areas, making it difficult to constrain a large number of sliding distribution parameters^12^, whereas InSAR data are more abundant. Data from two InSAR instruments, Sentinel‐1 and ALOS‐2, were accessed following the Mw6.4 and Mw7.1 Ridgecrest earthquakes on July 4 and 6, 2019, respectively. Due to the long operation cycle of InSAR satellite, it is difficult to distinguish coseismic versus post-earthquake deformation information, among which all InSAR data have been directly applied to inversion fault-slip distribution^2,13,14^. Sentinel‐1 data have been used separately to invert fault-slip distributions^12,15^. The various fault divisions employed in previous research, especially the different settings for fault dip parameters and types of data, have resulted in variations in the distribution of slip; that is, the maximum slip of the Mw7.1 seismic fault range from 3 to 6 m, whereas the maximum slip of the Mw6.4 seismic fault ranges from 0.6 to 1.0 m^12–15^. Therefore, to obtain a more accurate seismic fault-slip distribution, we used the precise positioning results and surface fracture conditions of the Ridgecrest area from January 2019 to October 2020 to distinguish eight faults for inversion of the fault dip. The fault dip angle was determined by seismic clustering, and then establish the fault geometric model of the area, including the latitude and longitude of the start and end points of the faults, as well as their respective strike and dip. Based on this model, we use differential InSAR (D-InSAR) data of the three coseismic orbits in the area and the Steepest Descent Method (SDM)^21^ for inversion of the slip distribution of the two seismic faults, and then compared our results with those of other research groups. Finally, the impacts of InSAR-derived dip data and other factors on the inversion are discussed, and the conclusions were presented.

## Data

### InSAR data

**Figure 1:**
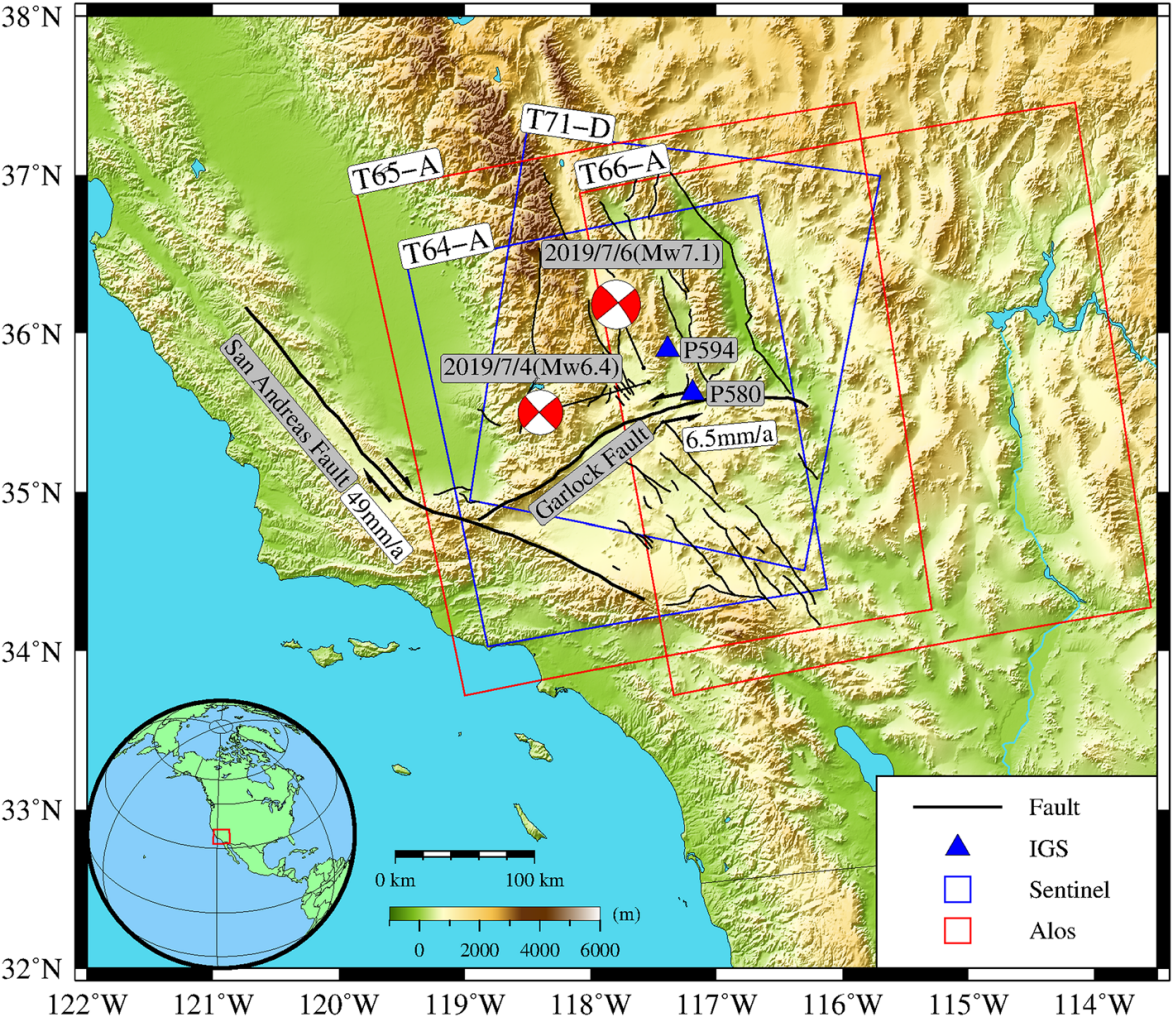
Tectonic setting of the 2019 Ridgecrest earthquake sequence and International GNSS Service (IGS) station location and InSAR data coverage.

In this study, we used InSAR data processed by Xu et al.^17^ published in Seismological Research Letters and publicly available at https://topex.ucsd.edu/SV_7.1/20. Data provided by Sentinel-1, a c-band synthetic aperture radar satellite operated by the European Space Agency, and ALOS-2, an L-band satellite operated by the Japan Aerospace Exploration Agency, were used to prepare a total of four Line of sight (LOS) aligned interferograms. In Fig. 1, the blue rectangular-outlined areas covered by the Sentinel-1 images were named as T64-A and T71-D, red rectangular-outlined areas covered by the ALOS-2 images were named as T65-A and T66-A, and two red focal mechanisms depict the fault plane solutions of the Mw 6.4 foreshock and Mw 7.1 mainshock. The orbital parameters are shown in Table 1. On the T64-A, T65-A and T66-A tracks, the maximum observed line-of-sight shortening were 62, 66 and 92 cm, respectively, and the maximum line-of-sight extension of the three-ascending orbit interferograms were 71, 93 and 65 cm, respectively.

**Table 1:** Parameters of interferometric pairs of Sentinel-1/ALOS-2 InSAR images.

Sensor	Track (A/D)	Master (YMD)	Slave (YMD)	Look angle (°)	Azimuth angle (°)	Perpendicular baseline (m)
Sentinel-1A/1B	T64A	20190704	20190710	36.5–41.8	− 10	133.13
Sentinel-1A	T71D	20190704	20190716	36.6–41.7	− 170	− 30.45
ALOS-2	T65A	20160808	20190708	26.1–31.9	− 10	− 2.4
ALOS-2	T66A	20170812	20190713	36.5–41.5	− 10	− 14.6

*A or D denotes ascending or descending, respectively.

The two red focal mechanisms delineate fault plane solutions of the Mw 6.4 foreshock and Mw 7.1 main shock (https://www.globalcmt.org/CMTsearch.html). The locations of IGS sites are marked by blue triangles. The blue rectangles outline the spatial coverage of the Sentinel-1 images (T64-A, T71-A) and red rectangles outline the spatial coverage of the ALOS-2 images (T65-A, T66-A). Black lines are active faults. The inset on the lower left shows the location of the study area. The drawing use Generic Mapping Tools 5.0 (GMT 5.0). Image overlain on the 30 m Shuttle Radar Topography Mission Digital Elevation Database.

**Figure 2:**
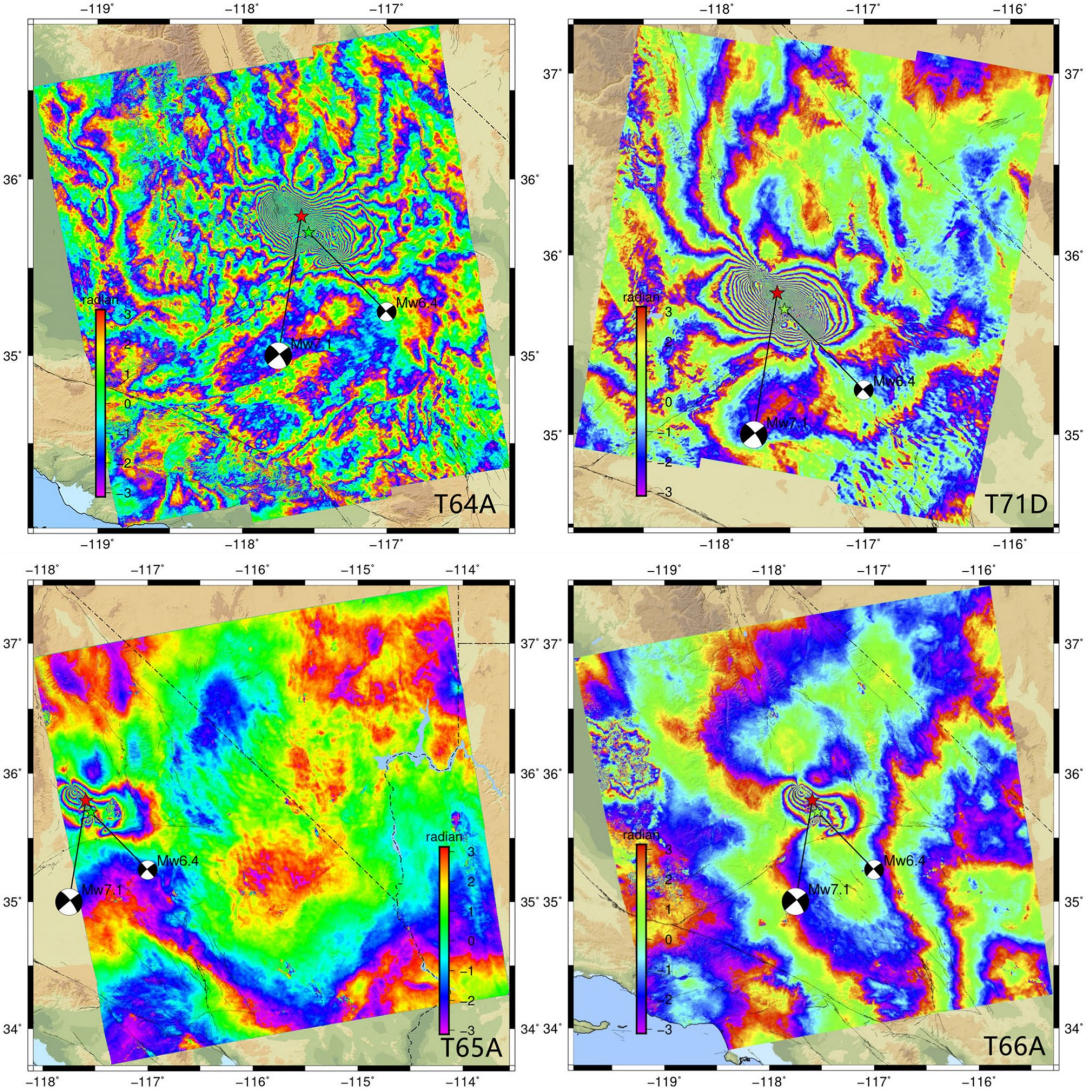
Different orbital phase diagrams^17^ (https://topex.ucsd.edu/SV_7.1/).

As shown in Fig. 2, the InSAR data were processed with open source software GMTSAR and mapped using GMT. For the Sentinel-1 interferogram maps, each fringe represents 2.8 cm of ground displacement away from the satellite. For the ALOS-2 maps, each fringe represents 12 cm of ground displacement.

### Small-earthquake data

Historically, a large number of small earthquakes have occurred in and near fault planes. It is assumed that a plane can be used to simulate the seismogenic fault, and most small earthquakes occur near this plane. This study used precise positioning data of the Ridgecrest area from January 2019 to October 2020 to establish the fault network structure of the region.

Black circles are small earthquakes of Mw > 1.0 from the United States Geological Survey (USGS) catalog (https://doi.org/10.5066/P91WN1UQ). The white lines indicate the simplified faults. Gray lines represent surface ruptures mapped by the USGS. Blue and red lines indicate active faults.

**Figure 3:**
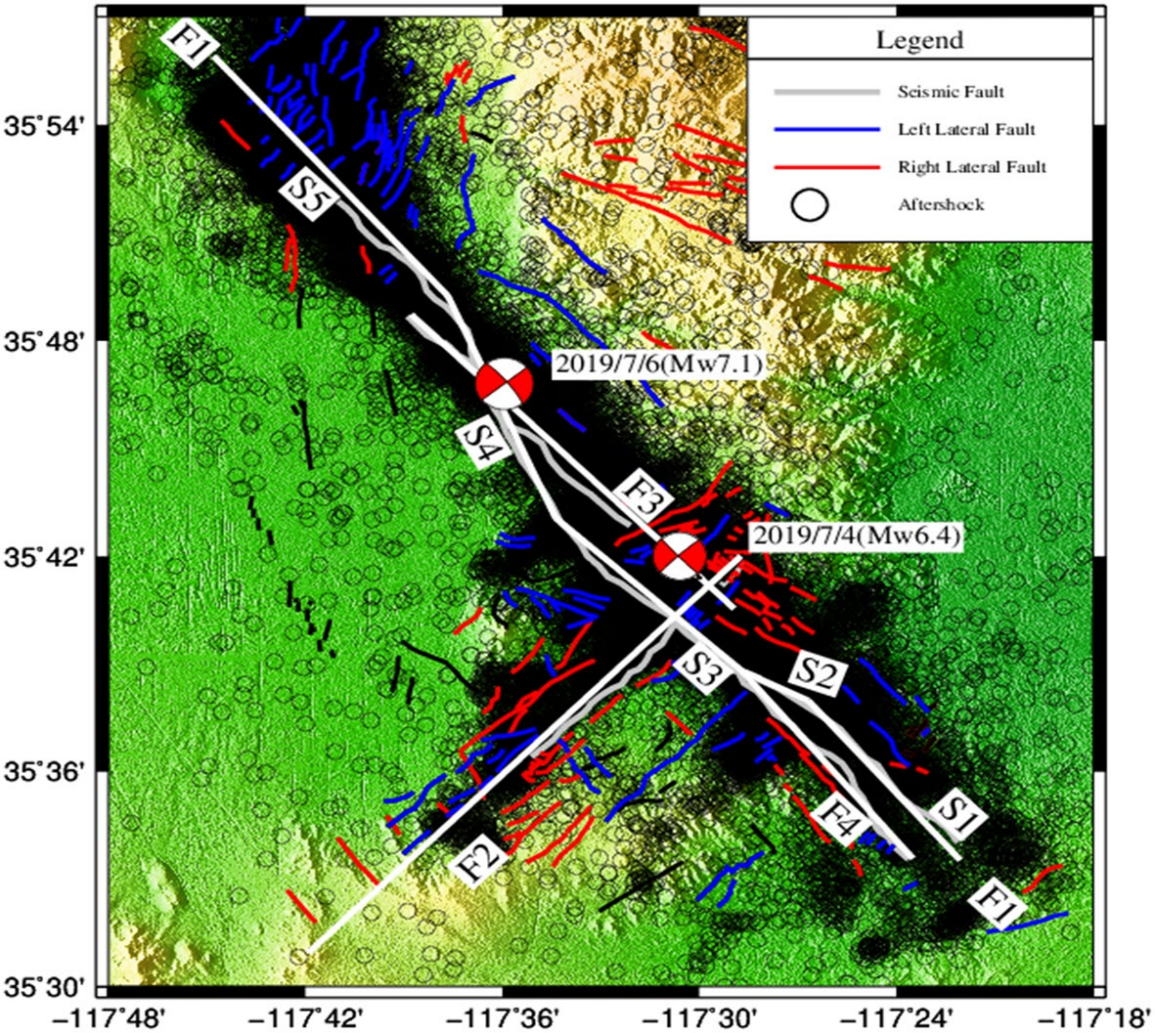
Distribution of small earthquakes and simplified fault model.

In Fig. 3, there were 23,151 small earthquakes with a magnitude greater than 1.0. The small-earthquake data included time, longitude, latitude, focal depth, magnitude, and focal mechanism solutions, which can solve the strike, dip and position of the seismogenic fault and the data obtained has a high precision^22^. The Libcomcat software was used to download the United States Geological Survey (USGS) Earthquake Catalog (https://doi.org/10.5066/P91WN1UQ). Seismic Coordinates are given in the WGS84 reference frame. The hypoDD method was used to relocate the focal location of small earthquakes. The absolute horizontal error of small-earthquake positioning in the catalog was less than 0.75 km, and the vertical error was less than 1.25 km. Therefore, the overall dataset shows reasonable accuracy^16^. The maximum depth of small earthquakes was 30 km, and the maximum magnitude was 5.5.

**Figure 4:**
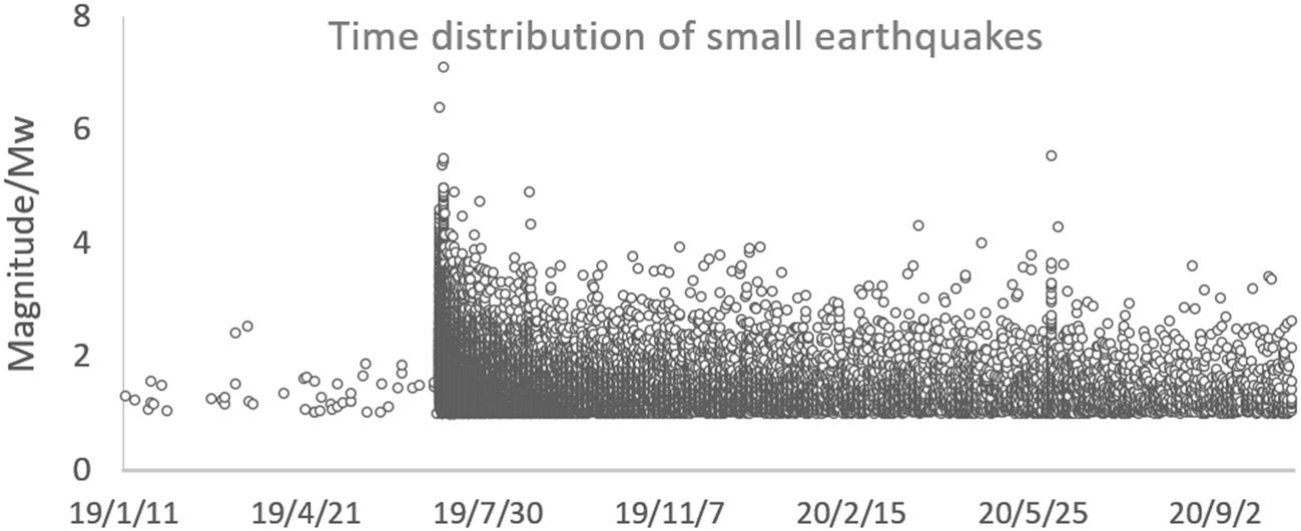
Time series diagram of small-earthquake.

Figure 4 shows the time series of small earthquakes with magnitudes greater than Mw1.0. The focal depths were mainly distributed at 0–15 km, accounting for 99.58% of all focal points; the magnitudes of the small earthquakes were mainly in the range of 1–5, accounting for 78.49% of all focal points.

## Methods

### Calculation of fault dip

Inversion of the fault-slip distribution must be based on the fault geometric model to include the coordinates of the start and end points, strike, and dip of the fault as the geometric parameters. First, we simplified the active fault fracture marks on the surface into segments of solid or broken lines. In Fig. 3, the gray fault is the trajectory of the fault’s projection onto the surface^14,23^. For more convenient calculation, the surface projection was simplified into four white faults, namely F1, F2, F3 and F4. Among these, the F1 fault varied greatly along strike so was subdivided into five sub-faults: S1, S2, S3, S4 and S5. Next, the dip of each fault was estimated based on the principle of small-earthquake clustering. The specific method involves taking the midpoint of the fault as the reference point and making a normal at that point that is perpendicular to the fault strike and pointing in the direction of the dip. Next, we use the horizontal normal (central axis) at the reference point as the horizontal axis and the depth as the vertical axis, then project all earthquake focuses within a certain distance from the central axis onto this plane^18,19^. We fitted the fault dip onto the plane based on the distribution of earthquakes. The maximum likelihood method was used to determine the dip of the fault. The specific calculation is described as follows:

1 $$\text{F}\left({\updelta }\right)=\prod _{i=1}^{n}\left\{\left[\frac{1}{{\sigma }_{i}\sqrt{2\pi }}{\exp}\left\{-\frac{{\left\{\left[{u}_{i}-x\left(\delta \right)\right]\times {\cos}\left({\updelta }\right)\right\}}^{2}}{2{{\sigma }_{i}}^{2}}\right\}\right]{\omega }_{i}+\omega \right\}$$

F(δ) describes the difference distance from the ground point to the fault plane determined by the two methods, where ${\sigma }_{i}$ is the variance of the probability density function obeyed by the focal depth of the seismic earthquake. Here, we assume that the error of each seismic earthquakes obeys a normal distribution; $x\left(\delta \right)$ is the theoretical focal depth; $x\left(\delta \right)\times$ cos $\left(\delta \right)$ is the distance from the ground point(focal point on the ground projection) to the fault surface; ${u}_{i}$ is the reported depth of the earthquake; ${u}_{i}\times$ cos(δ), the same as above; ${\omega }_{i}$ is the weight of the earthquake, which is the square of the magnitude of the earthquake; and ω is a parameter used to prevent the first item in the large parentheses from approaching zero, with the value typically set to 0.1.

To verify the credibility of linear plane fitting, residual analysis and fitting correlation analysis of the fitting were performed. The equation for residual calculation is:

2 $$\text{RMS}\left({\updelta }\right)=\frac{\sum _{i=1}^{n}{{\omega }_{i}\left\{\left[{u}_{i}-x\left(\delta \right)\right]\times {\cos}\left({\updelta }\right)\right\}}^{2}}{\sum _{i=1}^{n}{\omega }_{i}}$$

The equation for calculating the fitting correlation is:

3 $${\text{R}}^{2}=1.0-\frac{\sum _{i=1}^{n}{{\omega }_{i}\left\{\left[{u}_{i}-x\left(\delta \right)\right]\times {\cos}\left({\updelta }\right)\right\}}^{2}}{\sum _{i=1}^{\text{n}}{{\upomega }}_{i}{\left({u}_{i}-\stackrel{-}{u}\right)}^{2}}$$

### Slip distribution

Under normal circumstances, the epicenter model used for inversion of the slip distribution of the fault fracture is a half-space elastic dislocation model. The input parameters of the dislocation model are divided into fault geometric parameters (coordinates of the fault center, strike, dip, length, width and depth) and slip parameters (strike-slip, dip-slip and open). If the slip distribution on the fault plane is uneven, the fault plane needs to be divided into multiple sub-fault planes. The combination of the slip parameters of each sub-fault plane constitutes the slip distribution of the entire fault fracture.

The slip momentum smoothing condition means that the slip momentum on the current subfault plane and slip momentum on the surrounding subfault plane are continuous or only slightly change. The stress-drop smoothing condition means that the stress drop on the current subfault plane and the stress drop on the surrounding subfault plane are continuous or only slightly change. The stress drop of the current sub-fault is determined by the slip momentum of the fault, the depth of the fault and the shear modulus of the surrounding medium.

The inversion model is expressed in Eq. (4):

4 $${\left\|W(Gs\pm d)\right\|}^{2}=min$$

where $G$ is the Green’s function of the surface deformation caused by unit slip; $s$ is the slip; $d$ is the observed value of surface displacement; and $W$ is the weight of the observed value.

## Results

### Fault dip

Based on the distribution map of the active structure and level of seismic activity, the faults in the study area were first simplified to include only eight faults (Fig. 3). Second, small earthquakes less than 6 km from the fault (except for S1 and F5, which were set to 3 km) and all small earthquakes within a 10 km range from the central axis, were projected onto the plane. Considering the number of earthquakes and positioning errors, the starting magnitude was Mw 1.0. Using the distribution of earthquakes on each fault listed in the earthquake catalog, and considering the north and south dips of faults with an east–west strike, the dip range was set to 0–180°. Equations (1), (2) and (3) were used to determine the dip of each fault (see Table 2).

**Table 2:** Fault geometry parameters.

Fault	Fault parameters of Feng et al.^13^ Width (km)/strike (°)/dip (°)	Fault parameters of Li et al.^14^ Width(km)/strike (°)/dip (°)	Fault parameters in this study Width (km)/strike (°)/dip (°)
S1	15/134.2/89	30/320/83	20/320/88
S2	15/130.3/100	30/297/83	20/297/89
S3	15/140.9/93	30/313/83	20/313/89
S4	15/150.9/95	30/337/83	20/337/86
S5	15/140.1/93	30/318/83	20/318/87
F2	15/226.1/78	30/225/81	20/225/88
F3	–	30/316/90	20/316/84
F4	15/144.6/95	30/320/90	20/320/84

**Figure 5:**
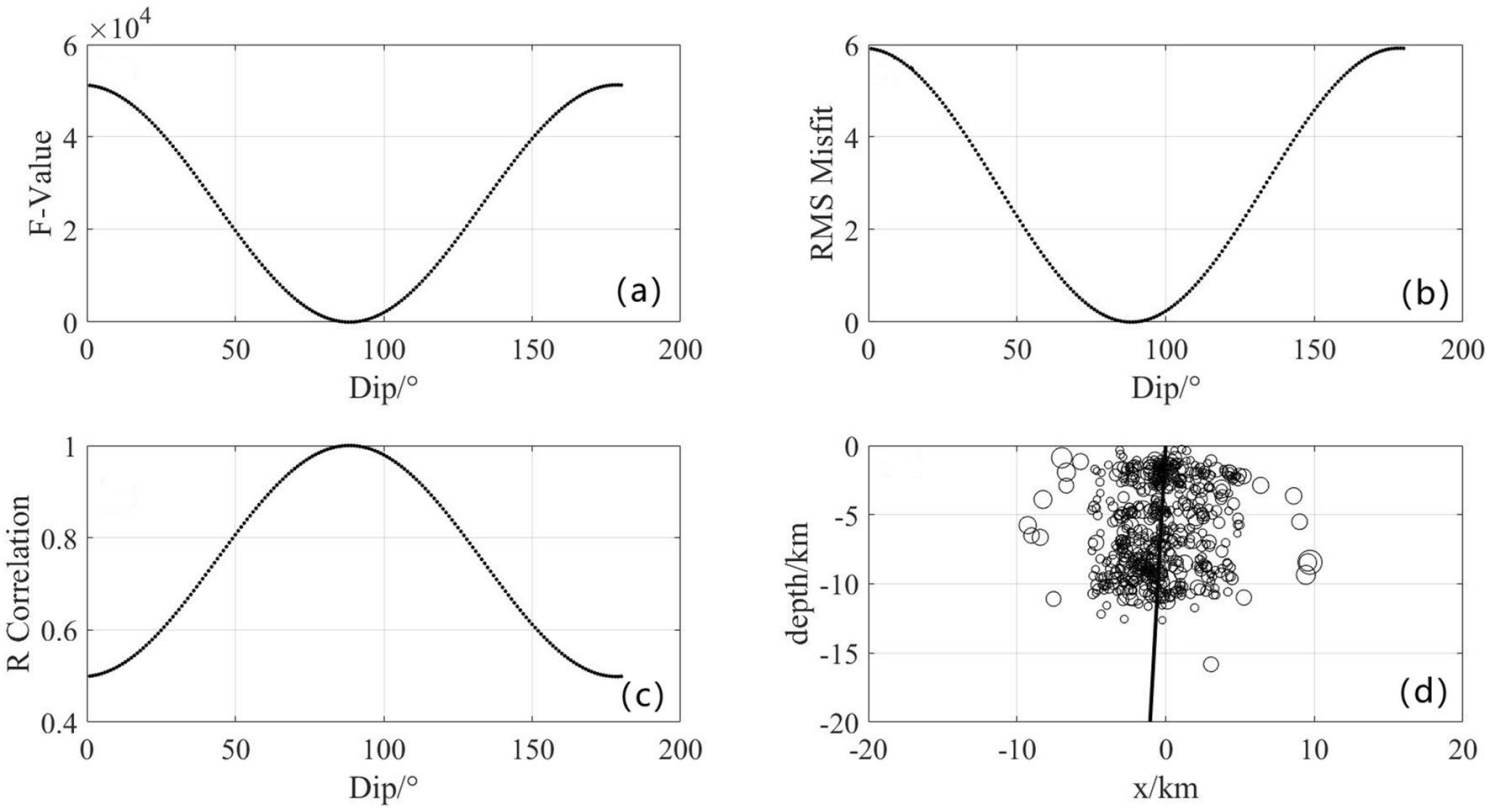
Dip calculation results of fault F2.

Taking "F2 fault" as an example, Figure 5(a) represents the mismatch between the objective function and dip-angle; (b) represents the fitting residual between the objective function and dip-angle; (c) represents the fitting correlation between the objective function and dip-angle; (d) represents the profile of the distribution of faults and small earthquakes with the horizontal axis showing the distance perpendicular to the fault strike and the vertical axis showing the depth, the small circle represents the location of the small earthquake, the magnitude represents the magnitude, the black line represents the position of the fault in the section.

When the fit of the objective function $\text{F}\left({\updelta }\right)$ reached the maximum, the dip ${\updelta }$ represented the best result. In Fig. 5, when the dip was 88°, the mismatch of the objective function $\text{F}\left({\updelta }\right)$ reached a minimum of − 1.8 × $10^{3}$, the fitting residual reaches a minimum of − 1.2, and the fitting correlation reached a maximum of 1.095. These results indicate the high credibility of the fitting results.

In this study, the fault width was set to 20 km. According to Fig. 3, small earthquakes were mainly distributed at a depth of 0–20 km, and the width was between 15^13^ and 30 km^14^. To study the differences in the fault dip parameters, we made the division of sub-faults and their strikes consistent with that of Li et al.^14^. A comparison with Table 2 shows that the dips of S1, S3, S4 and S5 of the F1 fault and the dip of the F4 fault were approximately the same as that stated in Feng et al.^13^, with a maximum difference of 3°. In addition, these two results were greater than the dip of the various faults in Li et al.^14^ (constant = 83°). For S2 of the F1 fault, the maximum difference between the dip derived in this study and that of Feng et al.^13^ was 9°. This was mainly because the difference in strike during fault division was 13.3°. The dip of the F2 fault derived in this study was larger than all the results of previous studies, i.e., the difference from Feng et al.^13^ was 10° and that from Li et al.^14^was 7°. The difference between the dip of the F3 fault derived in this study and that reported by Feng et al.^13^ was 6°.

### Inversion of the fault-slip distribution

**Figure 6:**
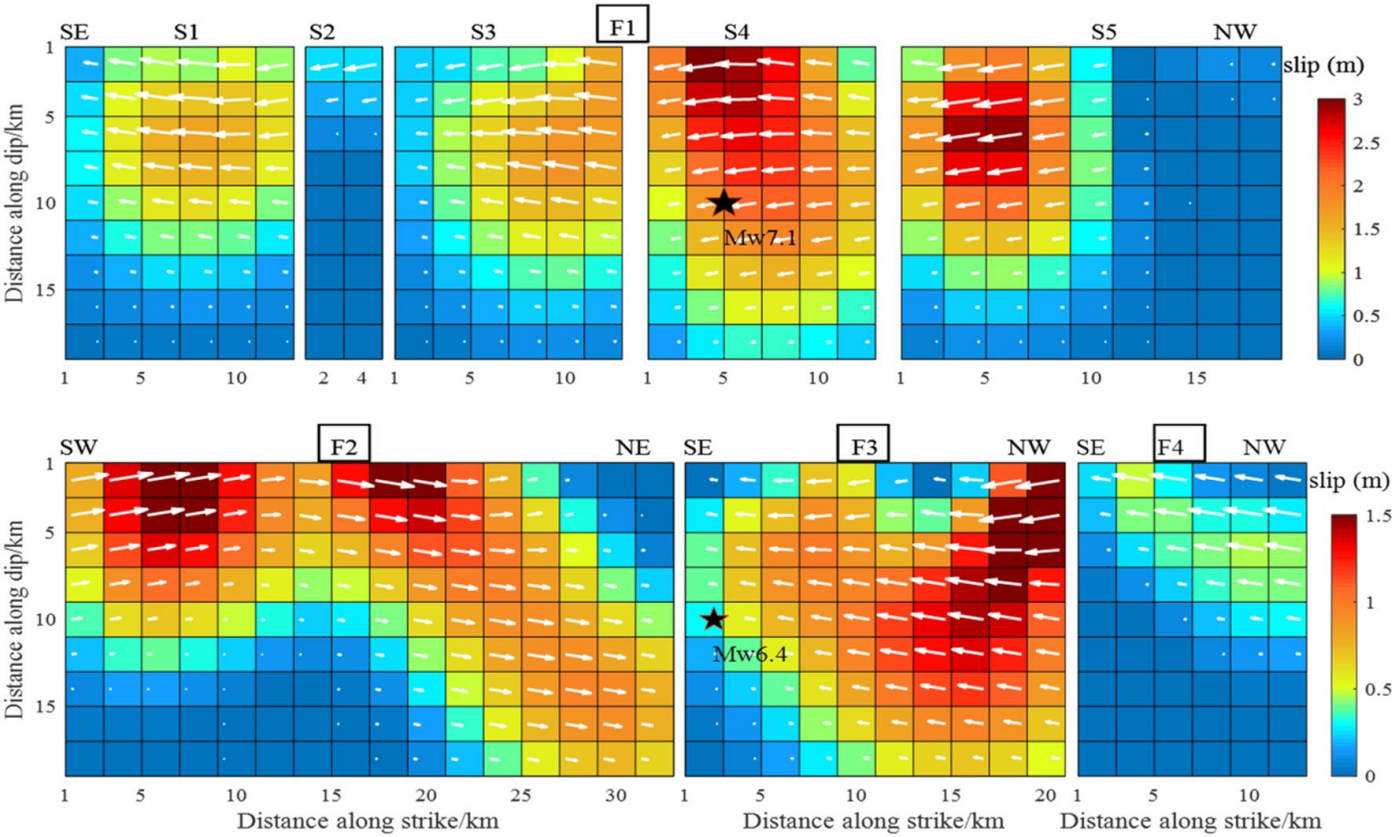
The slip distribution of the Mw 6.4 foreshock and the Mw 7.1 main shock from inversion of InSAR coseismic displacements.

The width of the eight faults was set to 20 km based on the fault dip parameters determined above. The faults were divided into 2 $\times$ 2 km planes, giving a total of 392 sub-faults. We used 5744 InSAR data points from three orbits, including 1924 T64-A data, 1896 T65-A data and 1924 T66-A data. The weight ratio, set based on residual analysis of the data orbit, was T64-A:T65-A:T66-A = 1:2.5:1. Using the SDM inversion program^21^, the slip was selected to satisfy the constraints of Laplace smoothing. The Laplacian second-order difference operator was used to construct the slip smoothing matrix of fault distribution. In this paper, the Laplace smoothing matrix was constructed by using four adjacent rectangular fault slices with equal angles and distances. Inversion of the earthquake slip distribution is shown in Fig. 6.

The first row from left to right, corresponds to the slip distribution of sub-faults S1, S2, S3, S4 and S5, respectively. The second row corresponds to the slip distribution of F2, F3 and F4. In all panels, On the sub-faults, the magnitude of the slip is indicated by a chroma bar and the slide angle by a white arrow. The black stars are the epicenters from USGS. These designations are used in subsequent figures.

The overall fault-slip distribution depth was 0–12 km. Apart from F2, which was a dextral strike-slip fault, all faults were sinistral strike-slip faults, with maximum slip of 3.0 m. The slip of the various faults typically extended along the direction of the fault strike, and the Euclidean vector along the fault width was close to zero. On the F1 fault, the two slip peaks were located on the S4 and S5 sub-faults at a depths of 2 km and 6 km, respectively. They were both above the Mw7.1 earthquake. Although the azimuth from the S1 to S5 sub-faults changed continuously, the change in the dip was minimal. The slip distribution was approximately continuous, especially in the S3, S4 and S5 sub-faults. The F3 fault intersected with the S4 sub-fault. The maximum slip of F3 was located 2.5 km above the NW end point, and the maximum slip was 1.5 m at a depth of 0–10 km. Compared with the other faults, the F4 fault exhibited less slip. The maximum slip was 0.6 m at a depth of 0–10 km. There were also two peaks on the F2 dextral strike-slip fault, and the maximum slip was 1.5 m. The peaks were 8 km and 20 km from the SW end point and close to the surface.

The slip distribution of the S1, S2, S3, S4 and S5 faults in this study was similar to that calculated by Li et al.^14^, published in Geophysical Journal International. This was reflected in the continuous distribution of S3, S4 and S5 and the similar maximum slip (all 3.0 m), with a maximum value of 3 m distributed at 0–5 km of S4. That was quite different from the maximum slip of 6.0 m reported by Feng et al.^13^. This difference was observed because we evaluated five sub-faults with a slip of 3.0 m, whereas Li et al.^14^ involved more than 10 sub-faults with a slip of 3.0 m they adopted T71-D data. In addition, the slip angle of Li et al.^14^ was 180°, showing a pure strike-slip property, whereas the slip angle in this study exhibited slight fluctuations around 180°, showing a mainly strike-slip with a small amount of dip-slip component property (white arrow in Fig. 6).

## Discussion

### Sensitivity analysis

**Figure 7:**
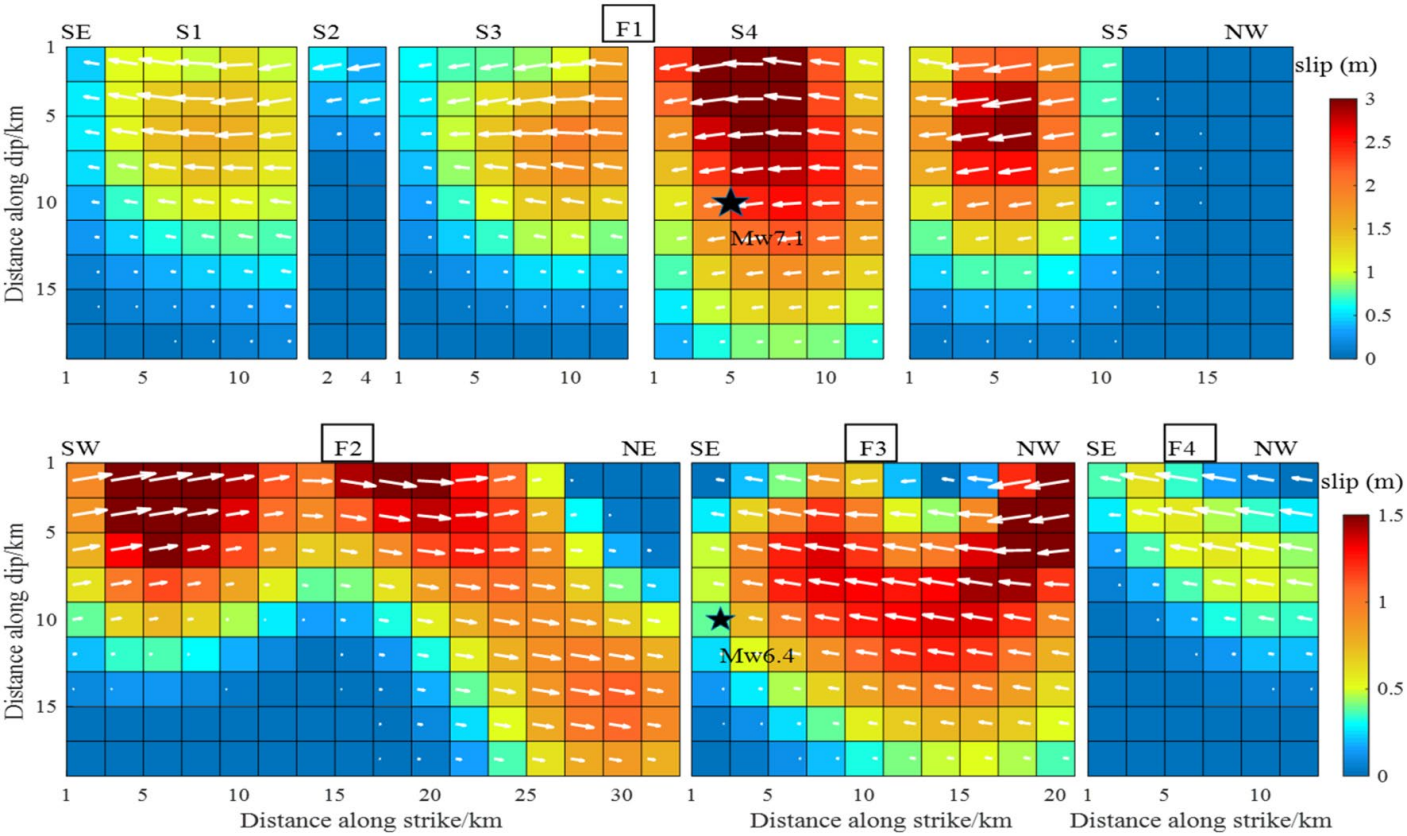
The slip distribution of the Mw 6.4 foreshock and the Mw 7.1 main shock from inversion of T64-A coseismic displacements.

**Figure 8:**
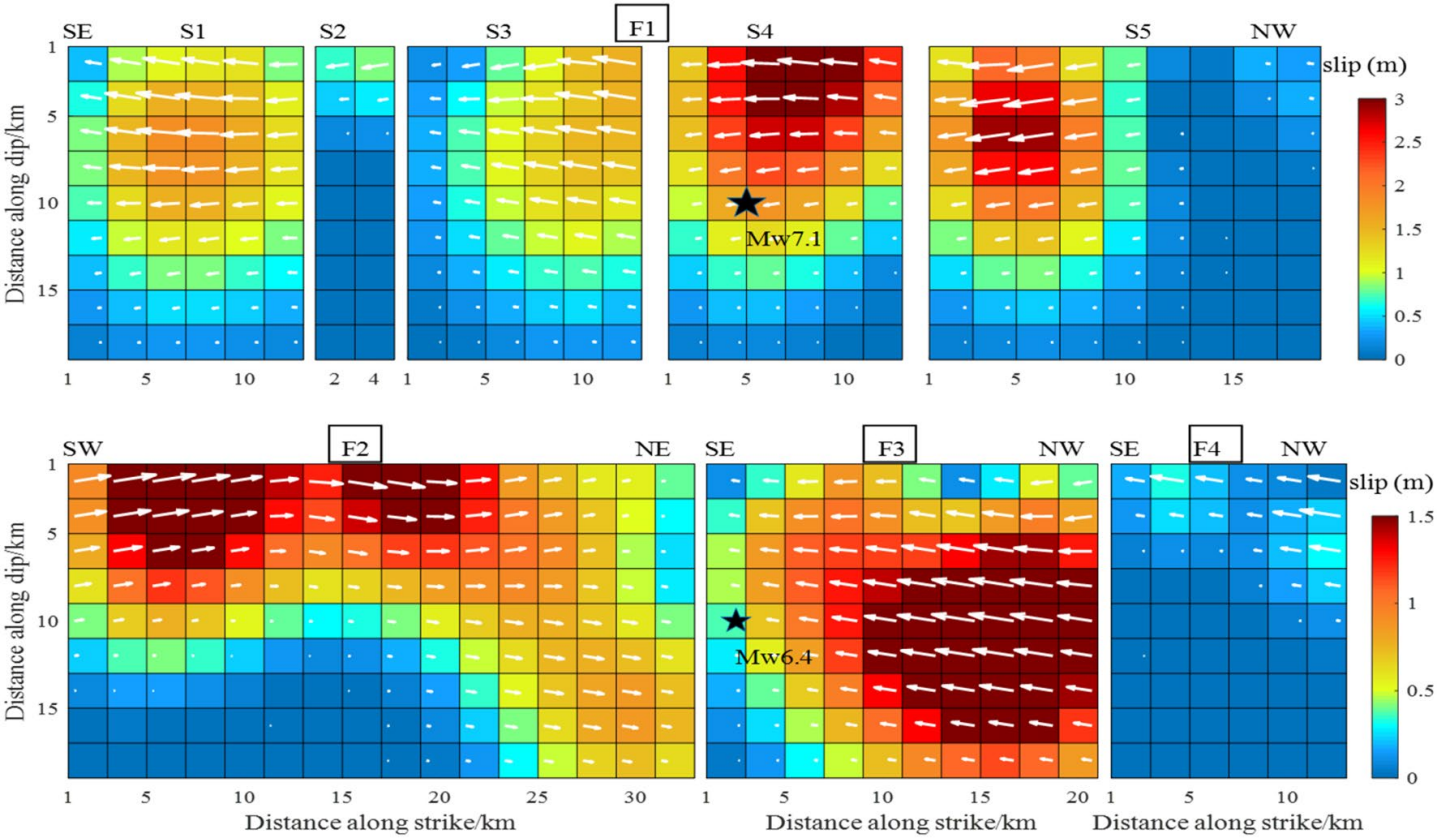
The slip distribution of the Mw 6.4 foreshock and the Mw 7.1 main shock from inversion of T65-A coseismic displacements.

**Figure 9:**
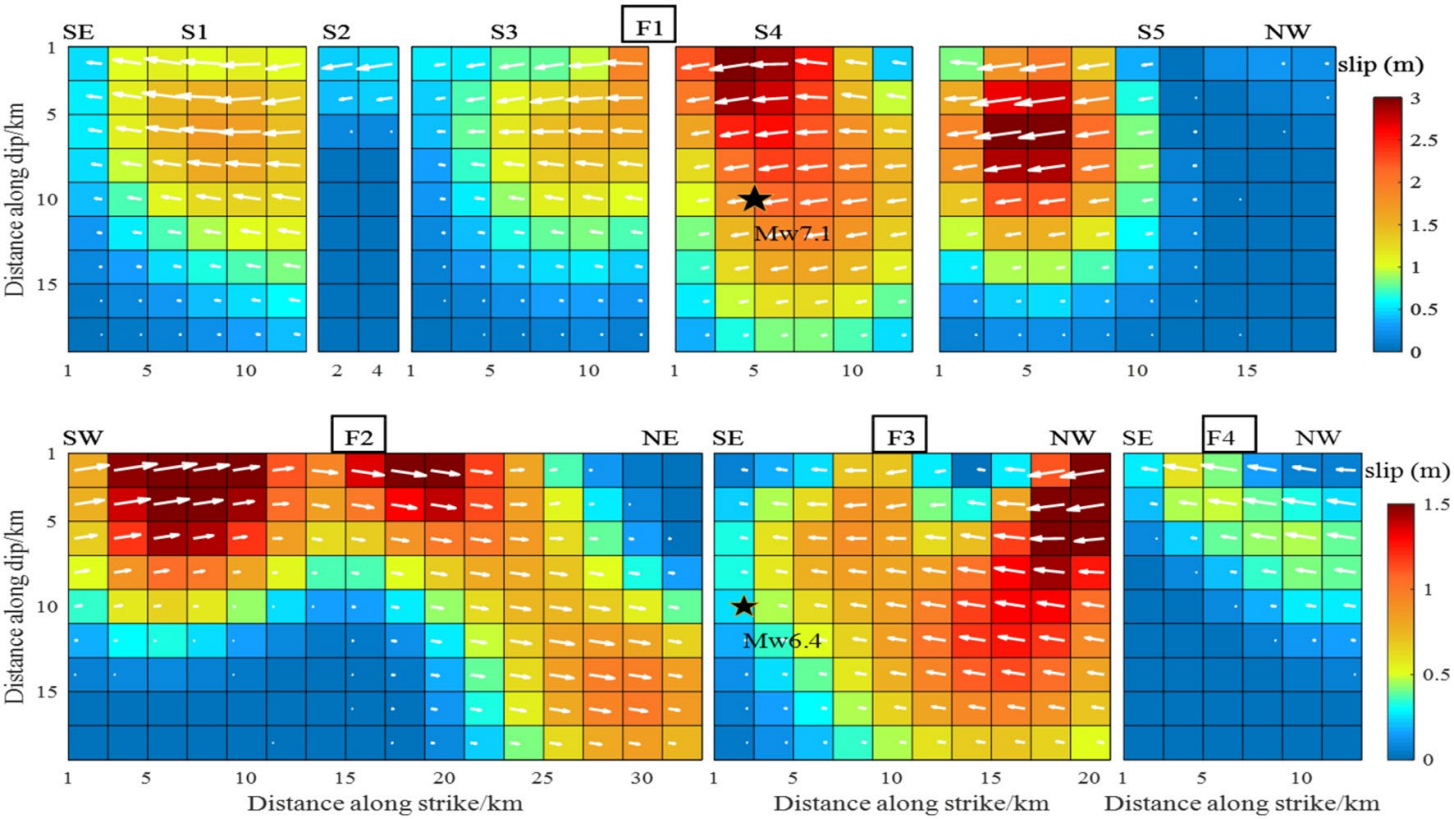
The slip distribution of the Mw 6.4 foreshock and the Mw 7.1 main shock from inversion of T66-A coseismic displacements.

**Figure 10:**
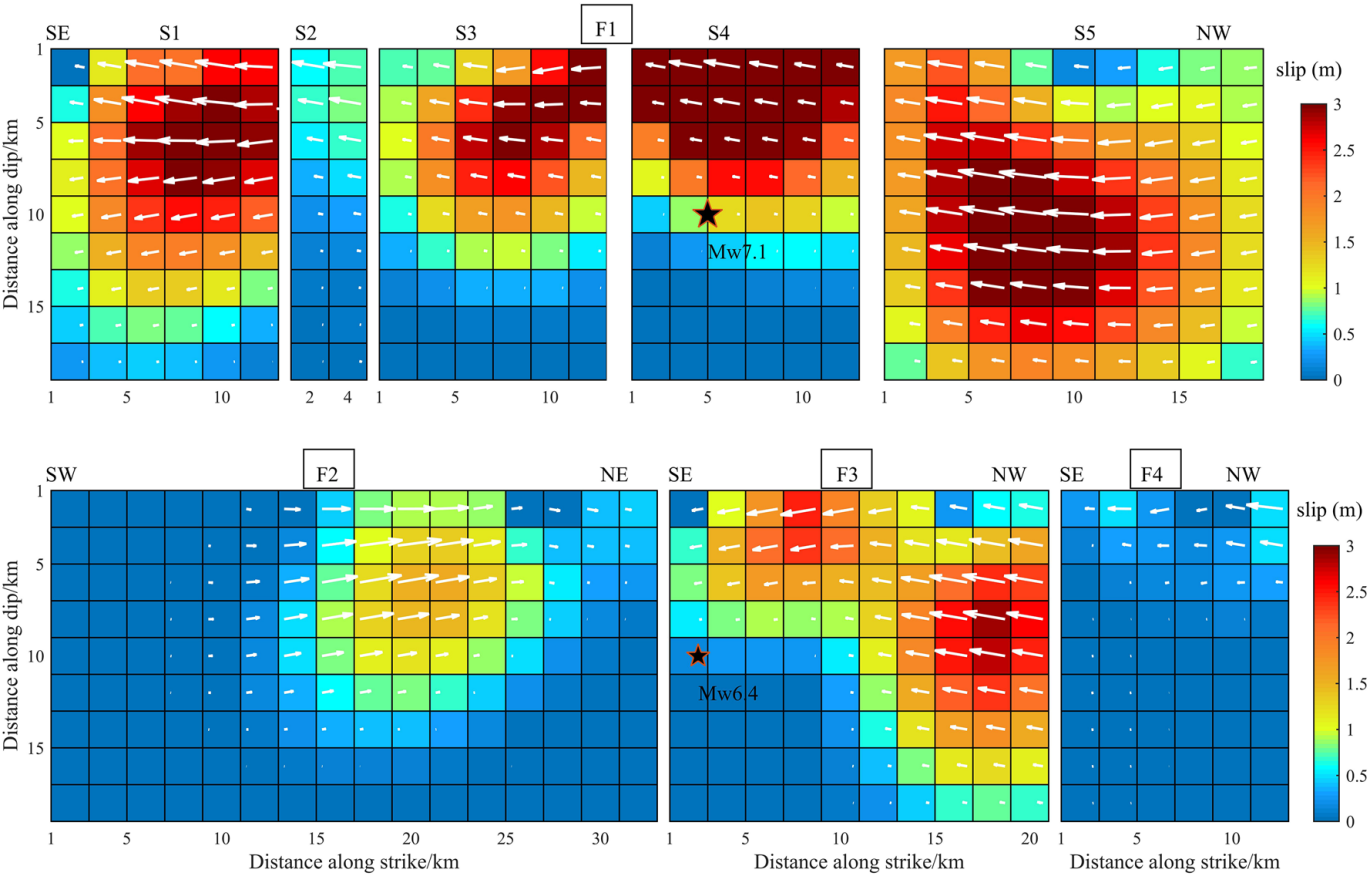
The slip distribution of the Mw 6.4 foreshock and the Mw 7.1 main shock from inversion of T71-D coseismic displacements.

In addition to the modeling factors, the data source was a key factor affecting the inversion results of the fault-slip distribution. This study used the InSAR data processed by Xu et al.^17^ of T64-A, T65-A, T66-A and T71-D orbits to perform inversion of the fault-slip distribution under similar conditions in terms of the geometric parameters and smoothing method. The results are shown in Figs. 7, 8, 9 and 10. Through comparative analysis, it was found that the slip distribution of different data inversions shared similar characteristics and the same magnitude. Nevertheless, certain variations were observed. When the four orbital data were used, all slip distributions were as follows: the slip depth was within 15 km; the maximum slip was on the S4 and S5 faults with a value of 3.0 m; and the slip distribution patterns from the inversion of T64-A, T65-A and T66-A data were the same, with 10, 9 and 8 rectangles reaching 3.0 m, respectively. There were two slip peaks on the F2 fault: the maximum value was 1.5 m, and the depth was less than 5 km. For the slip distribution inverted from T71-D, the maximum slip was reflected in the S1, S3, S4 and S5 sub-faults, as well as in the F3 fault. The number of rectangles reaching 3.0 m was 43, which was significantly more than the results derived using other data. The maximum slip of the S5 fault reached a depth of 15 km. However, there was only one slip peak on the F2 fault; its maximum magnitude was 1.8 m and the depth exceeded 5 km.

**Figure 11:**
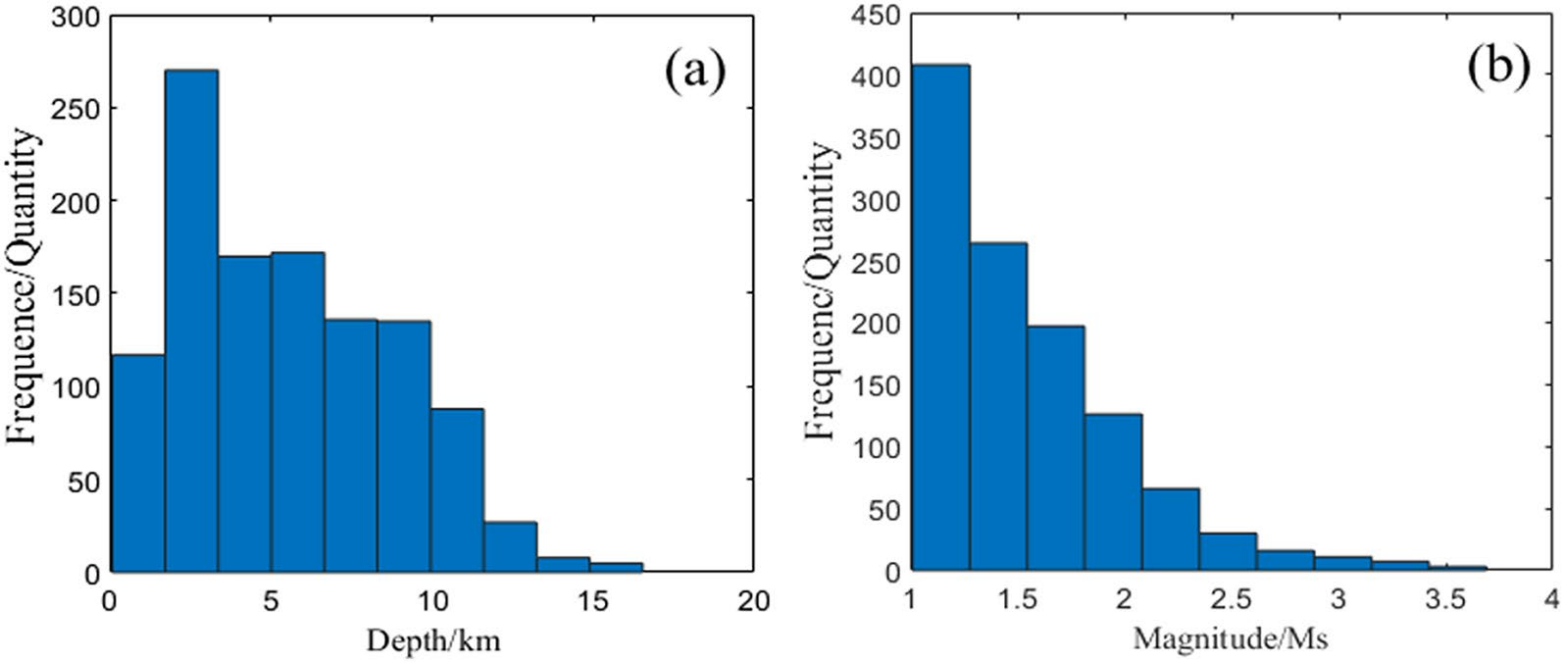
Small earthquakes from July 13, 2019 to July 16, 2019.

There are two reasons for the vast disparity between the fault-slip distributions inverted using T71-D data and other data. First, there were a total of 1,128 small earthquakes between July 13, 2019 and July 16, 2019. The depths and magnitudes of these earthquakes were 0–15 km and 1.0–3.8, respectively (see Fig. 11). In comparison, the revisit period of the T71-D data, from July 4–7,2019, was longer than the post-seismic duration included in the other data. The acquired data not only contained coseismic deformation, but also a large amount of information on post-seismic deformation. Second, the look angles for the first three orbits were 36°, 28° and 36°, respectively, whereas that for the T71-D orbit was 40°. The LOS data used for inversion is the synthesis of horizontal and vertical deformation components, and a large look angle is more sensitive to horizontal motion^24^, which may lead to the increased of LOS error.

**Figure 12:**
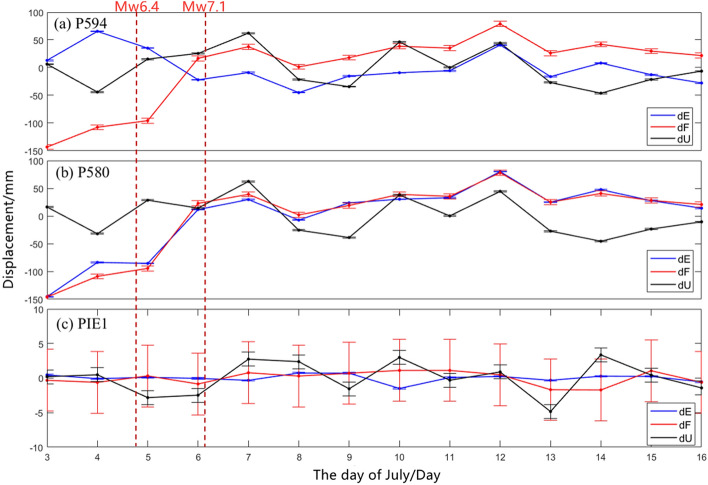
Site displacement calculated by IGS data.

To verify the impact of the after-earthquake deformation from July 13 2019 to July 16 2019 on surface, we used the Gamit 10.72 software^25^ to calculate the displacements of four IGS stations POVE, P594, P580 and PIE1 from July 3, 2019 to July 16, 2019. IGS can provide high-precision time-series deformation data, and the data (files on observed values, files on broadcast ephemeris, and files on precision ephemeris) were acquired from the IGS Data Center, Wuhan University (ftp://garner.ucsd.edu/archive/garner/rinex). The distances of the stations P580, P594, PIE1 and POVE from epicenter are 25 km, 30 km, 900 km and 2000 km. The POVE and PIE1 stations were taken as the reference stations because it was further from the earthquake area and cannot be shown in Fig. 1. For the P594 and P580 stations, the displacement of the 14-day solution results were – 150 to 90 mm, with a maximum of 150 mm (July 3, 2019). The GLOBK module was used to process the NEU coordinates of the various stations, and the average of the 14-day NEU coordinates of each station was taken as the reference value to obtain their respective daily residuals. These were separately projected onto the strike of the vertical fault, along the direction of the fault strike, and in the upward direction of the fault strike. We mainly analyzed the situation of P594, P580 and PIE1 stations, and the calculated displacement of the stations were shown in Fig. 12.

Figure 12(a) is the displacement of the P594 station, (b) is the displacement of the P580 station, and (c) is the displacement of the PIE1 station, the vertical bar in the figure is the error bar. The errors in dE, dF and dU directions of three stations are all less than 5 mm. The red dotted line is the occurrence time of the two earthquakes.

There were large changes in the dF, dE and dU vectors of the P594 and P580 stations.The most obvious changes was that from July 3 to July 7, the dF and dE components of P580 changed from − 150 to + 10 mm, which was mainly affected by two earthquakes. From July 7 to July 12, dF, dE and dU of the two measurement points were relatively stable and varied around 0 mm. From July 12 to July 16, dF, dE and dU components began to fluctuate, and the dF component of P594 decreased from 50 mm to about 0 mm, the dU of both stations decreased from 0 to − 50 mm first, and then recovered to around 0 mm. These data fluctuations were approximately consistent with the aftershock activities from July 13, 2019 to July 16, 2019, so the postseismic data should be avoided in the inversion calculation of fault slip distribution.

**Figure 13:**
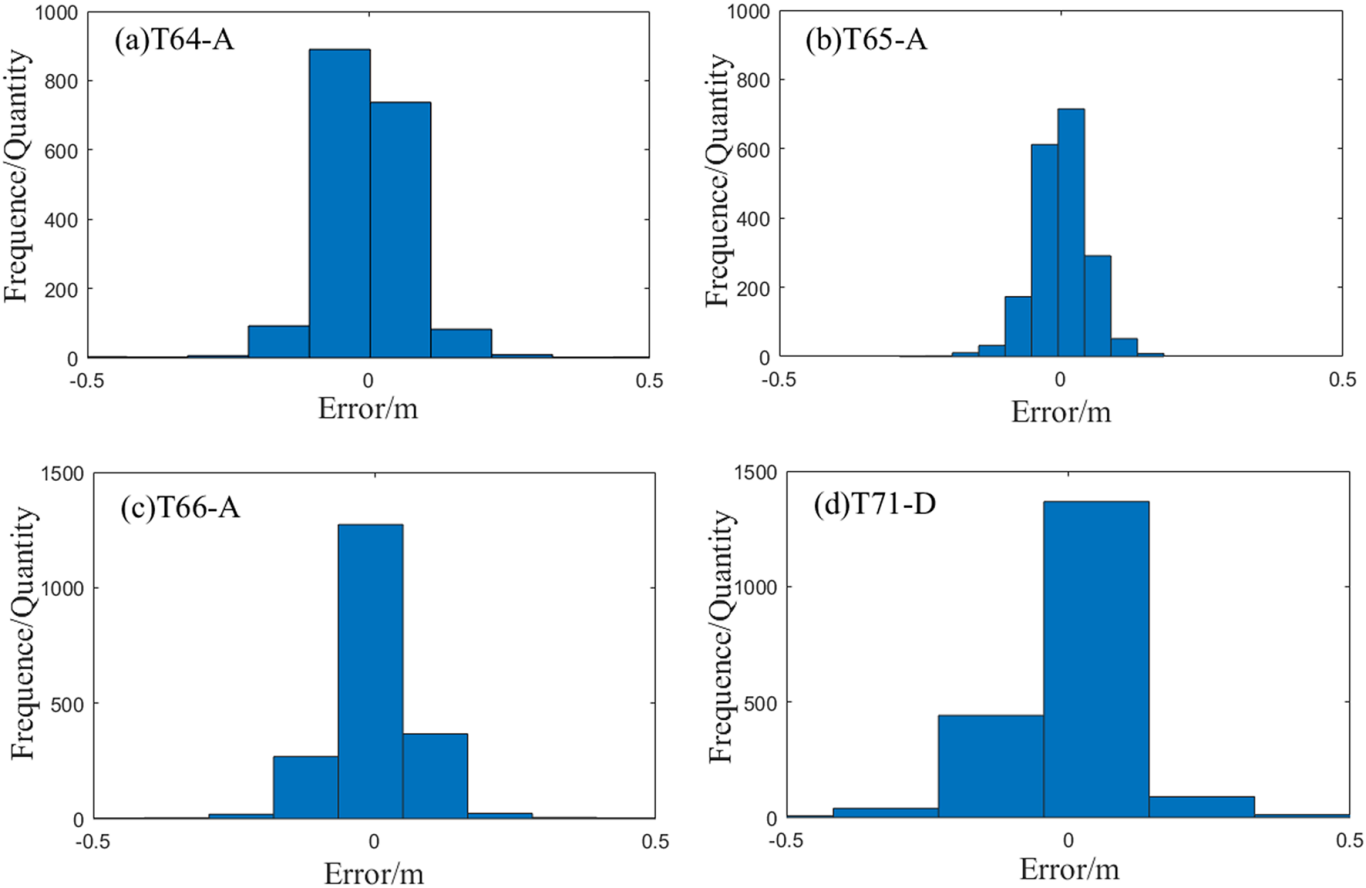
Statistical analysis of residuals.

To analyze the accuracy of the inversions of the slip distribution using the various orbital data and to determine the weight ratio of the inversion data, we performed a statistical analysis of the residuals of each orbit, as shown in Fig. 13(a)–(d) respectively represent the orbital residual of T64-A, T65-A, T66-A and T71-D. The residuals of the four orbital data were close to the normal distribution, the expected value of the residual was close to zero, and the maximum residual error did not exceed 0.48. The length of the T71-D residual model was the largest, occupying almost the entire − 0.5 to 0.5 interval. The length of the T65 residual model was the smallest; the number of residual data in the − 0.05 to 0.05 interval exceeded 60% of the total amount of data. After calculation, the standard deviations were 0.0751 m, 0.0488 m, 0.0773 m and 0.1137 m, respectively. Therefore, during joint inversion, their weight ratios were determined as 1.00:2.36:0.95:0.43. Considering that T71-D contained a large amount of post-seismic deformation information, T71-D data were discarded.

### Optimal smoothing factor

**Figure 14:**
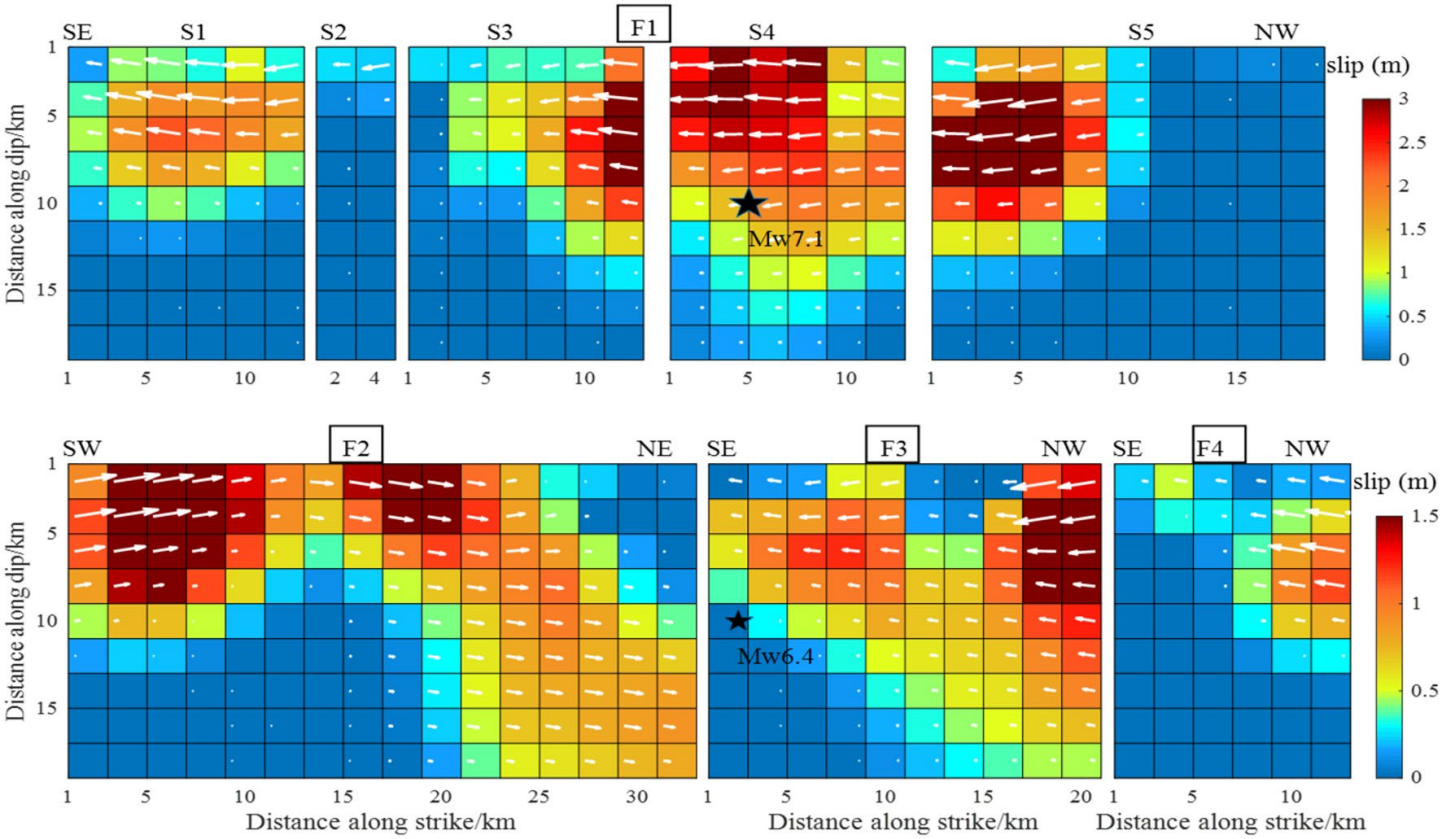
The slip distribution of the Mw 6.4 foreshock and the Mw 7.1 main shock by stress-drop smoothing condition.

We further compared the impact of the slip momentum smoothing condition and the stress-drop smoothing condition on the slip distribution. When the stress-drop smoothing condition was used, the maximum slip distribution was approximately 3.0 m and the corresponding depth was 8.0 km (Fig. 14). The maximum number of slip quantum sub-faults under the stress-drop smoothing condition (Fig. 14) was greater than that under the slip momentum smoothing condition (Fig. 6). For example, the maximum slip on the S5 sub-fault plane covered eight rectangles but only six rectangles in Fig. 6. There were thirteen rectangles on F2 sub-fault plane but only six rectangles in Fig. 6. Moreover, the results of the entire F1 fault were distributed along the fault in Fig. 14, causing notable leaps and oscillations. The standard deviation of the model’s fitting residual was 0.0784 m. If the slip momentum smoothing condition is used, these leaps in the slip distribution results can be avoided^26^, which was verified in this study. The maximum slip area and the significant slip area in Fig. 6 were similar to the results of previous studies^14^, and the standard deviation of the model’s fitting residual was 0.0736 m. Therefore, for these two earthquakes, we propose that the the slip momentum smoothing condition should be used as the constraint.

## Conclusions

The small-earthquake cluster that occurred in the Ridgecrest area from January 2019 to October 2020 was used to determine the dips of the S1, S2, S3, S4, S5, F2, F3 and F4 faults, which were 88°, 89°, 89°, 86°, 87°, 88°, 84° and 84°, respectively, enabling establishment of a fine fault geometry model for obtaining the slip distribution of the coseismic faults.Based on this, T64-A, T65-A and T66-A data were used for joint inversion of the slip distribution of the Mw6.4 and Mw7.1 coseismic faults.The results show that the slide angle is not exactly 180° but fluctuates slightly around this value. Apart from the dextral strike-slip F2 fault, all faults were sinistral strike-slip faults. On the F2 fault, the maximum slip was 1.5 m. Through experimental research, it was noted that it was more suitable to use the the slip momentum smoothing condition for inversion of the slip distribution of these earthquakes, rather than the stress-drop smoothing condition. In addition, we found that deformation on July 13–16, 2019 had a substantially impacted on the inversion results, which was confirmed by according to the displacement changes of the P594 and P580 IGS sites in the neighboring region of Ridgecrest. Therefore, this paper used T64-A, T65-A and T66-A data to jointly invert the slip distribution of the Mw6.4 and Mw7.1 seismic faults, providing a new fault-slip model for better interpretation of surface deformation.
