# Author Correction: Coseismic fault-slip distribution of the 2019 Ridgecrest Mw6.4 and Mw7.1 earthquakes

**DOI:** 10.1038/s41598-021-98294-0

**Published:** 2021-09-14

**Authors:** Yang Gao, HuRong Duan, YongZhi Zhang, JiaYing Chen, HeTing Jian, Rui Wu, WenHao Yin

**Affiliations:** 1 College of Geological Engineering and Geomatics, Chang’an University, Xi’an, 710064 Shaanxi China; 2 College of Geomatics, University of Science and Technology, Xi’an, 710054 Shaanxi China; 3 Xi’an Institute of Surveying and Mapping, Xi’an, 710054 Shaanxi China

Correction to: *Scientific Reports* 10.1038/s41598-021-93521-0, published online 09 July 2021

The original version of this Article contained errors.

In Figure 10, the slip color scale for Mw 6.4 foreshock (lower panel) was incorrect. As a result, the chroma subsampling was incorrect.

The original Figure 10 and accompanying legend appear below.

**Figure 10:**
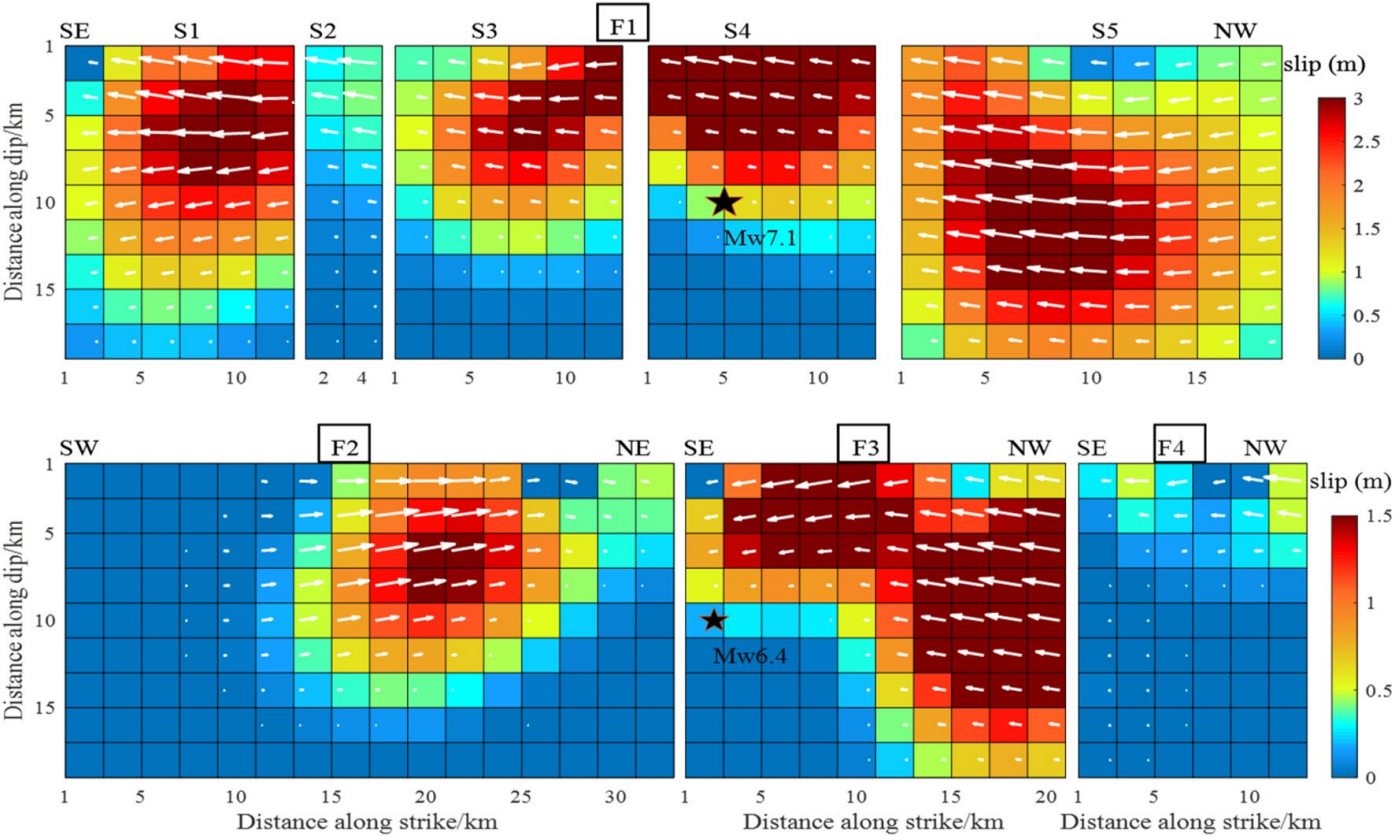
The slip distribution of the Mw 6.4 foreshock and the Mw 7.1 main shock from inversion of T71-D coseismic displacements.

Additionally, in the Conclusions section,

“On the F1 fault, The maximum slip was 1.5 m.”

now reads:

“On the F2 fault, the maximum slip was 1.5 m.”

The original Article has been corrected.

